# Virtual screening of inhibitors against Envelope glycoprotein of Chikungunya Virus: a drug repositioning approach

**DOI:** 10.6026/97320630015439

**Published:** 2019-06-15

**Authors:** Garima Agarwal, Sanjay Gupta, Reema Gabrani, Amita Gupta, Vijay Kumar Chaudhary, Vandana Gupta

**Affiliations:** 1 Center for Emerging Diseases, Department of Biotechnology, Jaypee Institute of Information Technology, Noida, UP 201309, India; 2 Centre for Innovation in Infectious Disease Research, Education and Training, University of Delhi South Campus, Benito Juarez Marg, New Delhi 110021, India; 3 Department of Microbiology, Ram Lal Anand College, University of Delhi South Campus (UDSC), Benito Juarez Marg, New Delhi 110021, India

**Keywords:** Chikungunya Virus (CHIKV), Drug repositioning, Structure-based virtual screening, CHIKV envelop glycoproteins

## Abstract

Chikungunya virus (CHIKV) a re-emerging mosquito-borne alpha virus causes significant distress which is further accentuated in the lack of specific therapeutics or a preventive vaccine, mandating accelerated research for anti-CHIKV therapeutics. In recent years, drug repositioning has gained recognition for the curative interventions for its cost and time efficacy. CHIKV envelope proteins are considered to be the promising targets for drug discovery because of their essential role in viral attachment and entry in the host cells. In the current study, we propose structure-based virtual screening of drug molecule on the crystal structure of mature Chikungunya envelope protein (PDB 3N41) using a library of FDA approved drug molecules. Several cephalosporin drugs docked successfully within two binding sites prepared at E1-E2 interface of CHIKV envelop protein complex with significantly low binding energies. Cefmenoxime, ceforanide, cefotetan, cefonicid sodium and cefpiramide were identified as top leads with a cumulative score of -67.67, -64.90, -63.78, -61.99, and - 61.77, forming electrostatic, hydrogen and hydrophobic bonds within both the binding sites. These shortlisted leads could be potential inhibitors of E1-E2 hetero dimer in CHIKV, hence might disrupt the integrity of envelope glycoprotein leading to loss of its ability to form mature viral particles and gain entry into the host.

## Background

Chikungunya virus (CHIKV), a mosquito-borne alphavirus, transmitted through Aedes aegypti and Aedes albopictus has become a global threat causing recurrent epidemic. With its first outbreak reported from Makonde, Tanzania in 1952, 1 it largely remained restricted to Africa and Asia, but in last few years, a large number of epidemic were also recorded from America as well as Europe 2. CHIKV fever is characterized by myalgia, polyarthralgia, fever, nausea, headache and skin rash 3. The word chikungunya means "to walk bent over" in the Makonde language of Africa in reference to the stooped posture acquired due to incapacitating arthralgia 4, that persist for months after acute infection is over.

CHIKV contains single-stranded, positive-sense RNA genome (11.8 Kb), with two open reading frames (ORFs). The 5' ORF encodes four non-structural proteins nsP 1-4, whereas the 3' ORF encodes five structural proteins, the capsid, 6K, and the envelope glycoproteins E1, E2, E3 3. E2 protein is responsible for the interaction with the host cell receptor whereas E1 mediates the fusion of the viral and host cell membrane during the viral entry process. E3 facilitates the formation of p62-E1 precursor complex 5, 6.

CHIKV has icosahedral symmetry presenting 80 spikes on its surface made up of glycoprotein E1 and E2 7. At neutral pH, E1 and E2 exist as heterodimers such that E1 lies below the E2. CHIKV enters the host cell by pH dependent receptor-mediated endocytosis, and in the acidic environment of the endosome, the complex of E1 and E2 heterodimer dissociates, leading to the formation of E1 homo-trimers. E1 mediates fusion between the viral and host cell membrane through its fusion peptide and releases capsid into the cytoplasm 8. During replication, structural proteins (p62 and E1) are transported to the plasma membrane via Golgi complex where p62 is cleaved into E2 and E3 7. The capsid protein present in the cytoplasm interacts with the E2 at plasma membrane catalyzing viral assembly 9 followed by the release of the mature viral particles.

The mature structure of CHIKV envelope glycoprotein reveals that interaction between the Glu50-Val60, Val229-Pro237 of E1 with Ala33-Arg38, Gln236-Arg244 of E2 play a crucial role in dissociation of E1-E2 heterodimer during viral entry. These residues together form a cavity on the surface that lies between E1-domain II and E2-β ribbon and also connects domain A to domain C of E2. In the low pH of the endosomal surrounding, these residues assist the conformational changes of E2 domain A with respect to domain B, resulting in the exposure of E1 fusion peptide. Besides this, the cavity looks like the mouth of the enzyme, and contains the allosteric site of furin proteases that cleave p62 into E2 and E3 during viral assembly. As a result, this cavity becomes very crucial, and binding of small molecule/ drug to this cavity possibly will hinder the viral entry as well as the assembly process 10.

Since CHIKV has become a major problem world over and the non availability of specific treatment and vaccine further complicates the situation. To block CHIKV at entry level, envelope glycoproteins are the possible targets for novel drug discovery. In the last few years, several studies have focused on the structure-based drug discovery. A variety of small molecules/ natural product libraries have been docked against CHIKV envelope glycoprotein; and molecules such as Chloroquine 11, Arbidol 12, Phenothiazines 13, Epigallocatechin gallate: a green tea component 14, Flavaglines 15, Obatoclax 16, Baicailin 17 were found to be potential inhibitors 18, 19, but none is yet approved for CHIKV treatment, hence there is a need to accelerate research to look for better and safer CHIKV inhibitors.

Drug repositioning or repurposing (proposing a second medical use of an already approved drug) has opened up new avenues in the therapeutic intervention 20, 21. Reduced time and cost for the discovery of new drugs makes it an attractive strategy for researchers working in the field of drug discovery. Many successful examples are there in the industry. One of them is sildenafil which was developed in 1989 and used for the treatment of angina, but now it is used in the treatment of erectile dysfunction and marketed as Viagra 22.

Molecular docking is the computational technique, which correctly predicts the interaction between receptor and ligands. Structure-based virtual screening of ligands can be done via docking of the library of ligands on receptors or docking sites prepared in proteins, resulting in a scoring function. The low binding score signifies higher affinity between the ligand and receptor 23, 24. In this study we propose, structure-based virtual screening of drug molecules currently used as the cell envelop inhibitors of bacteria, on the 3-D structure of mature envelope glycoproteins E1 and E2 of CHIKV with the aim to delineate potential novel inhibitors to restrict CHIKV entry into the host cells and also to inhibit viral assembly.

## Methodology

### Sequence retrieval and alignments:

The sequences of E1 and E2 proteins of various CHIKV strains were retrieved from protein database of NCBI. They were aligned using ClustalW with reference to the IND-06-GUJ strain of CHIKV.

### Receptor and ligand preparation:

The crystal structure of CHIKV envelope glycoprotein (E1-E2-E3) was retrieved from the Protein Data Bank (PDB ID: 3N41). Residues important for the formation of E1-E2 heterodimer were selected based on available literature. Two receptors (binding sites) were prepared around selected residues of B chain and F chain of the envelope glycoprotein (E1 and E2 glycoprotein respectively) that are conserved in almost all the strains of CHIKV with emphasis on Indian strains, using FlexX/LeadIT software. The structures of FDA approved drug molecules active on the cell wall and envelope of bacteria were obtained from the ZINC database in 3D Mol2 format.

### Molecular docking:

Molecular docking and structure based virtual screening were conducted using the FlexX/LeadIT software. In this study, the receptor is kept rigid while the flexible ligands are docked into it. This software is based on a robust incremental construction algorithm. The ligand is broken down into pieces and then flexibly docks on the active site of the receptor, using a variety of positional strategies. The poses are scored based on a variety of different scoring functions. The top ranked small molecules, as calculated using the binding energy scores in the FlexX software, were considered based on their binding pose and potential interactions with key residues. FDA approved drug library composed of 2924 compounds was screened for drug molecules that are effective on the bacterial cell wall. Selected 150 FDA approved drugs were docked into both the binding sites, and the resulting interactions were compared for best-fit drug molecules. The docking procedure was performed using the default settings.

### Analyzing and output visualization:

The hits were ranked according to their docking scores. The conformations with the lowest binding affinity were selected after the docking process and visualized using pose view of FlexX/LeadIT to analyze polar and hydrophobic bonds and to look for the interacting residues. These interactions were then further analyzed in detail along with the bond length determination using PyMol, and Discovery Studio. Further the poses and the interaction of the bound molecules were refined using energy minimization. Swiss PDB Viewer was used to perform Energy Minimization with the partial implementation of the GROMOS96 force-Field. The generated energy minimized poses were visualized using Pymol and Ligplot.

## Results and Discussion

**Figure 1:**
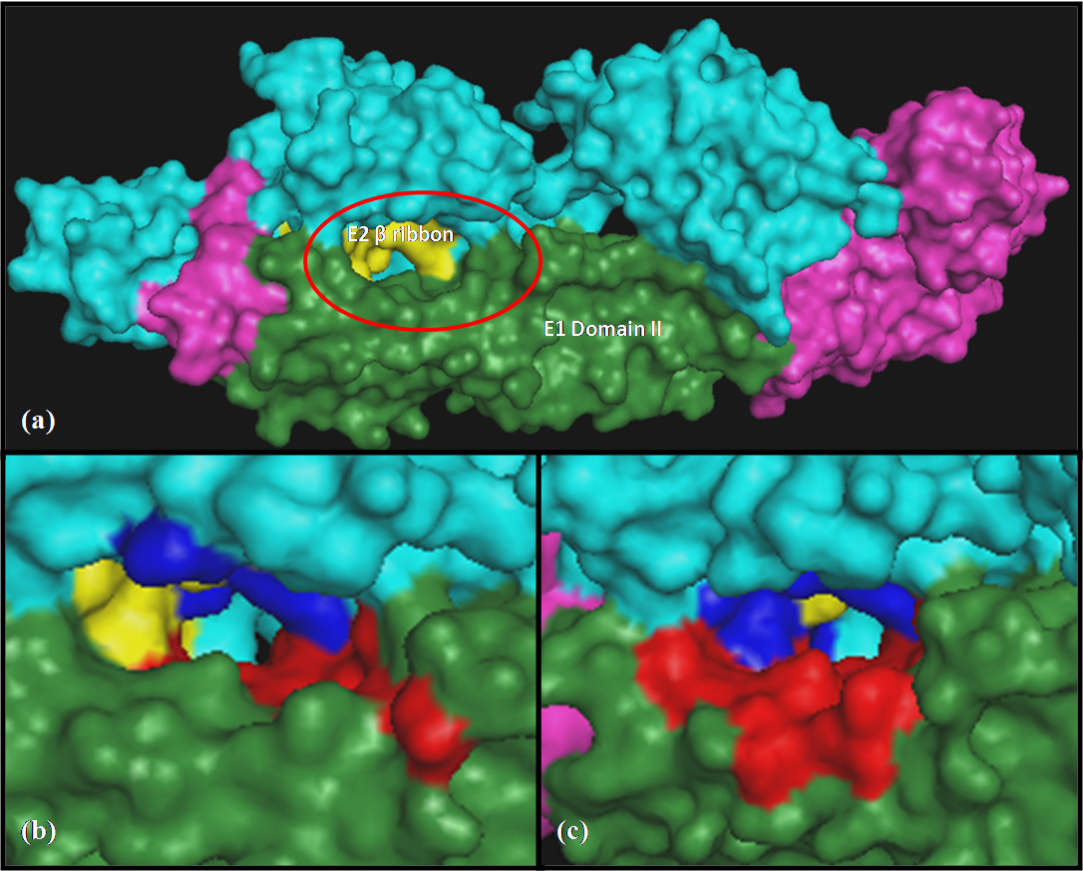
Three-dimensional structure of CHIKV envelope glycoproteins (3N41) where E1 and its domain II are shown in pink and green color similarly, E2 and its � Ribbon are shown in cyan and yellow color. Red and blue colors indicate the selected residues of E1 and E2 respectively that are involved in the formation of binding sites. (a) The selected region of the protein complex used for docking is encircled (b) location of binding site 1 (c) location of binding site 2

The accessible crystal structure and involvement of CHIKV envelope proteins in the viral entry and assembly process makes them apt for structure based drug designing. Though two forms of CHIKV envelope glycoproteins are seen in the cells, i.e. mature and immature and in this study we have chosen the mature envelope glycoprotein complex (spontaneously cleaved; 3N41) because it is present on the virion surface in the mature form. Crucial residues involved in E1 and E2 interaction were mined out from available literature and confirmed for their conservation by protein sequence alignments and were found to be conserved across almost all CHIKV strains particularly Indian strains (Data not shown). Using PyMol, the positions of these conserved residues were studied. It was observed that they combine to form a deep pocket between the domain II of E1 and the β ribbon of E2 protein (Figure 1a), where ligands can bind properly. Two binding sites were prepared from this base pocket (Figure 1b and Figure 1c) as it was big enough to conduct molecular docking. The residues of both the binding sites, which overlapped partially, are given in Table 1.

### Structure-based Virtual screening of the ligands:

From the docking studies, the top hits with the lowest scores were selected. Cephalosporins emerged as a major group of drugs/compounds that docked with significantly low score on both the binding sites. Top five common cephalosporins namely Cefmenoxime, Ceforanide, Cefotetan, Cefonicid sodium and Cefpiramide docked successfully with both the binding sites and were selected as leads (Table 2). Cephalosporin is a class of antibacterial drug that inhibits cell wall synthesis of bacteria, preventing cross-linkage of peptidoglycan by binding with penicillin-binding proteins (PBPs) or transpeptidases. They are highly resistant to hydrolysis by Beta-lactamases. Similar findings of inhibitory activities of cephalosporins have been reported earlier. Various cephalosporins have been reported to inhibit replication of herpes simplex virus I, vaccinia virus 25 and lentiviral RNase H 26. Recently inhibition of nsP3 helicase of HCV by cephalosporins is filed for Indian patent.

**Figure 2:**
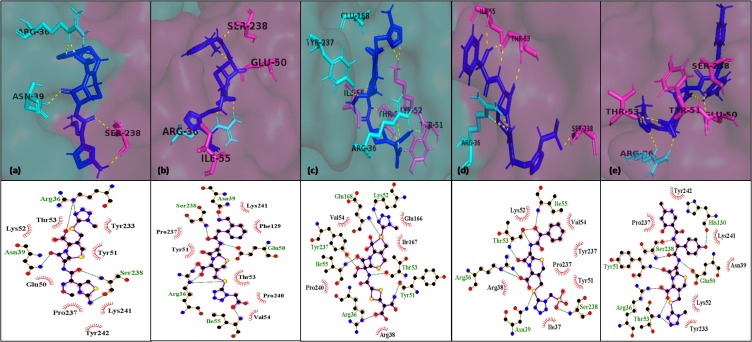
Interaction of selected leads with binding site 1 after energy minimization, (a) cefmenoxime (b) ceforanide (c) cefotetan (d) cefonicid sodium and (e) cefpiramide. E1 chain of the binding site is depicted as pink surface, E2 chain as cyan surface, their interacting residues as pink and cyan sticks respectively, ligand as blue sticks and polar contacts with interacting residues as yellow dotted lines. Also, their interactions are displayed in pose view

**Figure 3:**
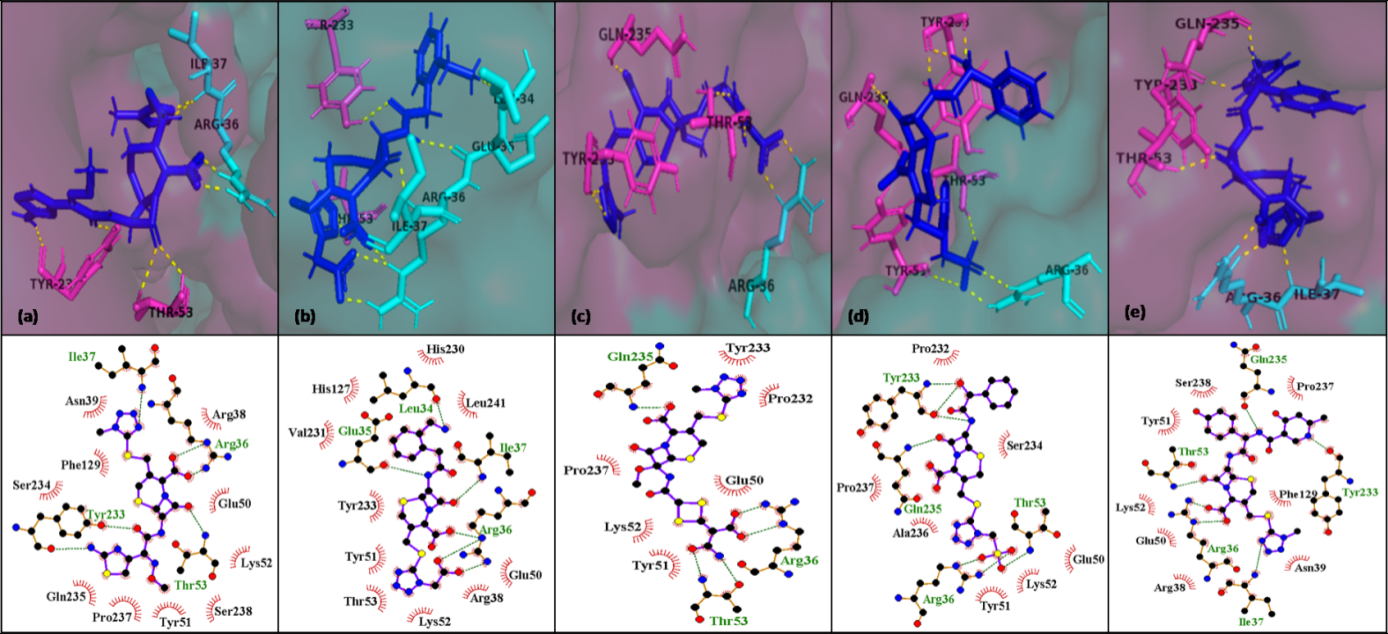
Interaction of selected leads with binding site 2 after energy minimization, (a) cefmenoxime (b) ceforanide (c) cefotetan (d) cefonicid sodium and (e) cefpiramide. E1 chain of the binding site is shown as pink surface, E2 chain as cyan surface, their interacting residues as pink and cyan sticks respectively, whereas ligands as blue stick H-bonding with interacting residues as yellow dotted lines. Also, their interactions are displayed in pose view

In our findings, among cephalosporin group, cefotetan has a better binding score with binding site 1 whereas cefmenoxime with binding site 2. But based on cumulative binding score cefmenoxime ranked first with the lowest cumulative binding score followed by ceforanide and others (Table 2). Ligands have been shown to make electrostatic, hydrogen and hydrophobic bonds with residues of interest as well as other residues within the pocket, which defines the uniqueness of the pocket, with a cumulative score as high as -67.67 (Table 2). We have performed the docking studies at multiple sites and selected common molecules exhibiting good scores at both the sites. This further enhances the significance of our results. The types of bonds they form, along with their bond lengths were analyzed using Discovery studio and the results are shown in the Table 3. The pose views of top five hits interacting with the residues of E1-E2 were refined by energy minimizations and visualized using PyMol and LigPlot (Figure 2 and Figure 3).

Arg36 and Ile37 in E2 protein, and Thr53, Tyr233, Gln235 and Ser238 in E1 protein are conserved in all Indian CHIKV strains and play an essential role in the formation of E1-E2 heterodimer 27, 10. These residues form hydrogen as well as hydrophobic bond with almost all the ligands adding significance to our docking results as leads interacting with these residues will have better chances of interfering with E1-E2 conformational changes during entry of CHIKV in the host cell and are worthy of further examination. Drug repurposing seems to be a laudable strategy for finding novel anti-CHIKV therapeutics as in a recent study it was reported that piperazine drug acts as a potential inhibitor of CHIKV by binding to the hydrophobic pocket of CHIKV capsid protein 28. A similar strategy is explored here for the disruption of E1-E2 interactions and we have shown that cephalosporins might acts as an anti-viral agent, which is unique with context to CHIKV envelope proteins. Although cephalosporins have been earlier reported to exhibit promising inhibitory activity against viruses such as herpes simplex virus I and vaccinia virus 25, but the work was not pursued further. Our results corroborate with these earlier reports and cephalosporin mediated interference with E1 and E2 heterodimer could lead to the inhibition of essential processes such as CHIKV entry as well as assembly in the host cell.

## Conclusion

Since there is an urgent need for anti-CHIKV drugs, repurposing of FDA approved drugs will be an excellent proposition as it will reduce the timeline for new drug discovery significantly. In our study, we could narrow down to five drug molecules namely cefmenoxime, ceforanide, cefotetan, cefonicid sodium and cefpiramide at in silico level all of which belong to the class cephalosporins, presently indicated for bacterial infections. Successful docking of these leads at two partially overlapping docking sites and their interaction with crucial conserved residues within the envelope protein of CHIKV further accentuate the implication of these results. The results are subject to further validation through in vitro and in vivo assays for inhibition of CHIKV entry and assembly.

## Conflict of Interest

The authors declare that they have no conflict of interest in the publication.

## Figures and Tables

**Table 1:** Residues involved in the formation of the binding sites

Proteins	E1	E2
Receptor 1	GLU50, TYR51, LYS52, THR53,	ARG36, PRO128, TYR237
	ILE55, SER238, TYR242	
Receptor 2	GLU50, TYR51, LYS52, THR53, VAL54, ILE55, PRO56, HIS230, VAL231, PRO232, TYR233, SER234, GLN235, ALA236, PRO237	ALA33, LEU34, GLU35, ARG36, ILE37, ARG38, ASN238, SER239, PRO240, LEU241

**Table 2:** Binding scores of top cephalosporin hits with both the receptors and their cumulative score

Ligands	FlexX score		Cumulative score
	Receptor 1	Receptor 2	
Cefmenoxime	-27.0932	-40.5769	-67.6701
Ceforanide	-28.7764	-36.1269	-64.9033
Cefotetan	-30.7352	-33.0493	-63.7845
Cefonicid sodium	-24.3021	-37.6939	-61.996
Cefpiramide	-22.5182	-39.2606	-61.7788

**Table 3:** Analysis of ligand-receptor interactions

Receptor 1			Receptor 2		
Residues	Bond type	Bond length (Å)	Residues	Bond type	Bond length (Å)
CEFMENOXIME					
E1:SER238	Conventional Hydrogen Bond	3.11	E1:THR53	Conventional Hydrogen Bond	2.85
E1:SER238	Conventional Hydrogen Bond	3.09	E1:THR53	Conventional Hydrogen Bond	3.22
E1:SER238	Conventional Hydrogen Bond	1.7	E1:TYR233	Conventional Hydrogen Bond	2.87
E1:GLU50	Carbon Hydrogen Bond	1.68	E1:TYR233	Conventional Hydrogen Bond	2.29
E1:GLU50	Carbon Hydrogen Bond	2.29	E1:PRO237	Carbon Hydrogen Bond	3.17
E1:TYR233	Carbon Hydrogen Bond	1.91	E1:TYR233	Carbon Hydrogen Bond	3
E1:TYR51	Hydrophobic: Pi-Alkyl	4.44	E1:THR53	Carbon Hydrogen Bond	2.49
E1:PRO237	Hydrophobic: Pi-Alkyl	4.4	E1:GLN235	Pi-Lone Pair	2.63
E1:LYS241	Hydrophobic: Pi-Alkyl	4.94	E1:PRO237	Hydrophobic: Pi-Alkyl	4.79
E2:ARG36	Conventional Hydrogen Bond	2.64	E2:ARG36	Electrostatic; Hydrogen bond: Salt Bridge	2.88
E2:ARG36	Conventional Hydrogen Bond	2.99	E2:ARG36	Conventional Hydrogen Bond	2.49
E2:ASN39	Conventional Hydrogen Bond	3.11	E2:ILE37	Conventional Hydrogen Bond	2.65
E2:ARG36	Electrostatic	2.91	E2:PHE129	Pi-Sulfur	4.5
E2:ARG36	Sulfur-X	3.33	E2:ARG36	Hydrophobic: Pi-Alkyl	4.93
E2:ARG36	Hydrophobic: Pi-Alkyl	3.75	E2:ILE37	Hydrophobic: Pi-Alkyl	4.64
E1:GLU50	Salt Bridge;Attractive Charge	2.3	E1:TYR233	Conventional Hydrogen Bond	3.39
E1:TYR51	Conventional Hydrogen Bond	2.89	E1:TYR233	Conventional Hydrogen Bond	2.66
E1:ILE55	Conventional Hydrogen Bond	2.63	E1:GLU50	Carbon Hydrogen Bond	2.86
E1:SER238	Conventional Hydrogen Bond	2.78	E1:TYR51	Carbon Hydrogen Bond	1.8
E1:GLU50	Conventional Hydrogen Bond	2.01	E1:TYR233	Hydrophobic: Pi-Sulfur	5.43
E1:THR53	Carbon Hydrogen Bond	2.23	E2:ARG36	Electrostatic; Hydrogen bond: Salt Bridge	2.78
E1:TYR51	Hydrophobic: Pi-Alkyl	5.23	E2:ARG36	Electrostatic: Attractive Charge	4.15
E2:ARG36	Attractive Charge	3.13	E2:GLU35	Electrostatic: Attractive Charge	4.24
E2:ARG36	Conventional Hydrogen Bond	2.53	E2:ARG36	Conventional Hydrogen Bond	3.1
E2:ARG36	Conventional Hydrogen Bond	2.53	E2:ARG36	Conventional Hydrogen Bond	2.97
E2:ASN39	Carbon Hydrogen Bond	2.32	E2:ILE37	Conventional Hydrogen Bond	2.81
E2:ARG36	Hydrophobic: Pi-Alkyl	4.12	E2:GLU35	Conventional Hydrogen Bond	2.23
			E2:LEU34	Conventional Hydrogen Bond	2.06
			E2:PRO240	Hydrophobic: Pi-Alkyl	4.79
			E2:LEU241	Hydrophobic: Pi-Alkyl	4.95
CEFOTETAN					
E1:LYS52	Electrostatic; Hydrogen bond: Salt Bridge	3.15	E1:THR53	Conventional Hydrogen Bond	2.9
E1:LYS52	Conventional Hydrogen Bond	2.35	E1:GLN235	Conventional Hydrogen Bond	2.77
E1:THR53	Conventional Hydrogen Bond	3.7	E1:THR53	Conventional Hydrogen Bond	2.25
E1:THR53	Conventional Hydrogen Bond	3.27	E1:PRO232	Carbon Hydrogen Bond	3.39
E1:ILE55	Conventional Hydrogen Bond	2.5	E1:TYR233	Hydrophobic: Pi-Alkyl	5.02
E1:THR53	Conventional Hydrogen Bond	1.93	E2:ARG36	Electrostatic; Hydrogen bond: Salt Bridge	2.85
E1:TYR51	Conventional Hydrogen Bond	1.84	E2:ARG36	Conventional Hydrogen Bond	2.93
E1:ILE55	Carbon Hydrogen Bond	2.33			
E1:THR53	Sulfur-X	2.99			
E1:GLU112	Electrostatic: Pi-Anion	4.93			
E2:ARG36	Electrostatic: Attractive Charge	5.51			
E2:ARG36	Conventional Hydrogen Bond	2.87			
E2:GLU168	Conventional Hydrogen Bond	2.7			
E2:TYR237	Conventional Hydrogen Bond	2.5			
E2:TYR237	Conventional Hydrogen Bond	2.67			
E2:GLU166	Carbon Hydrogen Bond	2.06			
E2:TYR237	Pi-Sulfur	5.04			
E2:ILE167	Hydrophobic: Alkyl	5.27			
CEFONICID SODIUM					
E1:TYR51	Conventional Hydrogen Bond	3.1	E1:TYR51	Conventional Hydrogen Bond	2.99
E1:THR53	Conventional Hydrogen Bond	2.7	E1:THR53	Conventional Hydrogen Bond	3.3
E1:SER238	Conventional Hydrogen Bond	2.81	E1:THR53	Conventional Hydrogen Bond	3.06
E1:SER238	Conventional Hydrogen Bond	2.87	E1:TYR233	Conventional Hydrogen Bond	2.56
E1:THR53	Conventional Hydrogen Bond	1.74	E1:GLN235	Conventional Hydrogen Bond	3.07
E1:THR53	Conventional Hydrogen Bond	1.66	E1:TYR233	Conventional Hydrogen Bond	2.11
E1:PRO237	Carbon Hydrogen Bond	3.21	E1:TYR233	Conventional Hydrogen Bond	1.68
E1:TYR51	Electrostatic: Pi-Anion	3.58	E1:TYR51	Carbon Hydrogen Bond	2.76
E1:LYS52	Hydrophobic: Pi-Alkyl	5.26	E2:ARG36	Electrostatic: Attractive Charge	3.73
E1:VAL54	Hydrophobic: Pi-Alkyl	5.03	E2:ARG36	Conventional Hydrogen Bond	3.09
E2:ARG36	Electrostatic: Attractive Charge	5.01	E2:ARG36	Conventional Hydrogen Bond	2.93
E2:ARG36	Conventional Hydrogen Bond	3.12			
E2:ARG36	Conventional Hydrogen Bond	3.03			
E2:ASN39	Conventional Hydrogen Bond	3.42			
E2:PHE129	Pi-Sulfur	5.82			
E2:ARG36	Hydrophobic: Alkyl	4.36			
CEFPIRAMIDE					
E1:TYR51	Conventional Hydrogen Bond	2.85	E1:THR53	Conventional Hydrogen Bond	3.01
E1:THR53	Conventional Hydrogen Bond	2.55	E1:THR53	Conventional Hydrogen Bond	3.2
E1:THR53	Conventional Hydrogen Bond	3.09	E1:TYR233	Conventional Hydrogen Bond	2.9
E1:SER238	Conventional Hydrogen Bond	2.44	E1:GLN235	Conventional Hydrogen Bond	2.17
E1:SER238	Conventional Hydrogen Bond	2.01	E1:TYR233	Conventional Hydrogen Bond	1.92
E1:GLU50	Conventional Hydrogen Bond	2.21	E1:TYR51	Carbon Hydrogen Bond	1.84
E1:LYS52	Carbon Hydrogen Bond	2.79	E1:THR53	Carbon Hydrogen Bond	2.86
E1:SER238	Carbon Hydrogen Bond	2.12	E1:TYR233	Carbon Hydrogen Bond	3.01
E1:TYR233	Carbon Hydrogen Bond	2.52	E1:SER238	Pi-Donor Hydrogen Bond	3.85
E1:TYR233	Carbon Hydrogen Bond	2.59	E1:TYR233	Hydrophobic: Pi-Pi T-shaped	5.76
E1:TYR51	Pi-Lone Pair	2.84	E1:PRO237	Hydrophobic: Pi-Alkyl	4.38
E1:TYR242	Hydrophobic: Pi-Pi Stacked	4.81	E2:ARG36	Electrostatic; Hydrogen bond: Salt Bridge	2.94
E1:PRO237	Hydrophobic: Pi-Alkyl	3.37	E2:ARG36	Conventional Hydrogen Bond	2.48
E1:LYS241	Hydrophobic: Pi-Alkyl	4.01	E2:ILE37	Conventional Hydrogen Bond	2.6
E1:LYS52	Hydrophobic: Pi-Alkyl	5.19	E2:PHE129	Pi-Sulfur	4.37
E2:ARG36	Electrostatic: Salt Bridge	2.68	E2:ARG36	Hydrophobic: Pi-Alkyl	4.77
E2:ARG36	Conventional Hydrogen Bond	3.15	E2:ILE37	Hydrophobic: Pi-Alkyl	4.83
E2:ASN39	Conventional Hydrogen Bond	2.89			
E2:ARG36	Hydrophobic: Pi-Alkyl	5.46
